# Perceived risks, concession travel pass access and everyday technology use for out-of-home participation: cross-sectional interviews among older people in the UK

**DOI:** 10.1186/s12877-020-01565-0

**Published:** 2020-06-05

**Authors:** Sophie Nadia Gaber, Louise Nygård, Anders Kottorp, Georgina Charlesworth, Sarah Wallcook, Camilla Malinowsky

**Affiliations:** 1 Department of Neurobiology, Care Sciences & Society (NVS), Division of Occupational Therapy, Karolinska Institutet, Fack 23 200, SE-141 83 Huddinge, Sweden; 2 Faculty of Brain Sciences, University College London, London, UK; 3 Faculty of Health and Society, Malmö University, Malmö, Sweden; 4 Research Department of Clinical, Educational, and Health Psychology, University College London, London, UK; 5 Research and Development, North East London NHS Foundation Trust, Ilford, UK

**Keywords:** Activities of daily living, Dementia, Environment, Older adults, Risk, Social participation, Technology

## Abstract

**Background:**

The health-promoting qualities of participation as an opportunity for social and cognitive engagement are well known. Use of Everyday Technology such as Smartphones or ATMs, as enabling or disabling factors for out-of-home participation is however under-researched, particularly among older people with and without dementia. Out-of-home participation involves participation in places and activities outside of a person’s home, in public space. Situated within the context of an increasingly technological society, the study investigated factors such as perceived risks, access to a concession travel pass and use of Everyday Technologies, and their relationship with out-of-home participation, among older people in the UK.

**Methods:**

One hundred twenty-eight older people with and without dementia in urban and rural environments in the UK, were interviewed using the Participation in ACTivities and Places OUTside Home (ACT-OUT) Questionnaire and the Everyday Technology Use Questionnaire (ETUQ). Associations between Everyday Technology use, perceived risk of falling, functional impairment, access to a concession travel pass and out-of-home participation were investigated using ordinal regression.

**Results:**

A higher probability of Everyday Technology use (Odds Ratio [OR] = 1.492; 95% Confidence Interval [CI] = 1.041–1.127), perceived risk of falling outside home (OR = 2.499; 95% CI = 1.235–5.053) and, access to a concession travel pass (OR = 3.943; 95% CI = 1.970–7.893) were associated with a higher level of out-of-home participation. However, other types of risk (getting lost; feeling stressed or embarrassed) were not associated with out-of-home participation. Having a functional impairment was associated with a low probability of a higher level of out-of-home participation (OR = .470; 95% CI = .181–1.223). Across the sample, ‘outside home’ Everyday Technologies were used to a higher degree than ‘portable’ Everyday Technologies which can be used both in and outside home.

**Conclusions:**

The study provides insights into perceived risks, access to a concession travel pass and use of Everyday Technologies, and their relationship with out-of-home participation, among older people in the UK. Increased knowledge about factors associated with out-of-home participation may help to guide targeted health and social care planning.

## Background

Participation particularly in cognitive and social activities is linked to health benefits which may prevent cognitive impairment, or decline, among older people at risk of developing dementia [1–3]. Whereas social isolation among older people living at home is a growing problem with serious implications for health and wellbeing [4]. Out-of-home participation refers to the way people participate in places and engage in meaningful activities outside their homes, in their communities [5]. As the incidence of people living in their ordinary homes, albeit with dementia or other age-associated disorders increases, there is a need to develop evidence about out-of-home participation among older people, with and without dementia [6].

Technological aspects of out-of-home participation are important to consider because there is evidence of older people using practices and routines to manage technologies for participation in everyday life [7]. ‘Full and effective participation and inclusion in society’ is a human right, prioritized by international legislation [8] and policy agendas [9]. Considering the potential health benefits associated with the right to participation and inclusion in society, it is necessary to investigate patterns of technology use in an everyday life context [7], referred to as Everyday Technology (ET) (e.g. Smartphone, Self-service checkout, Ticket machine for public transportation), as well as patterns of out-of-home participation among older people [10].

ET are ubiquitous across all locations that people perform activities in, both in and outside home. The proliferation of ET has however been accompanied by an increased expectation for being a skilled user of ET [11]. Such expectations can be problematic for older people because ET use requires numerous cognitive, perceptual and fine motor capacities, (e.g. memorizing and entering a PIN to make a card payment in a supermarket, or following steps to operate a ticket machine at a transportation center). Aging can impact skills necessary for performing activities of daily living using ET (e.g. due to changes in cognition, fine motor skills, or motivation) [12, 13]. Earlier studies indicate a potential association between lower involvement in activities outside home and use of fewer ETs among older people with cognitive impairments [14, 15]. This suggests that the increased challenges older people experience whilst using ET may be associated with decreased out-of-home participation, especially in those places predicated on ET use [11, 16].

A compensatory measure that may relieve the older person’s need to be a skilled user of ET is the concession travel pass (CTP). In recent years, a number of CTPs have become available as smart tickets, including the Free Older Person’s Bus Pass that enables free travel on local buses in England, the Senior Railcard which is an annual savings card for rail fares in the UK, and Transport for London’s Freedom Pass which provides free or discounted travel for London residents across London transportation networks [17]. Many CTPs are available to older people or those living with a disability. Access to a CTP may facilitate out-of-home participation using automated technology, without the need to access online payments or ticket machines [18]. However, earlier research has shown that it may be the subsidization itself that facilitates out-of-home participation through more accessible public transport. A potential association between access to a CTP and out-of-home participation warrants further investigation because studies show that access to a CTP is associated with health benefits among older people, including increased physical activity [19] and social engagement [20].

A review of the literature elucidates two factors which have not been thoroughly explored by existing research but which may have an enabling or disabling association with out-of-home participation: (i) functional impairment and, (ii) perceived risk [21]. A high proportion of older people are living with some type of functional impairment, including chronic disease, or multimorbidity, such as reduced fine motor skills and diabetes [22]. Functional impairment refers to the decline in a person’s ability to manage core activities of daily living, in addition to more complex instrumental activities of daily living. Instrumental activities of daily living can require out-of-home participation, such as managing financial tasks, using public transportation and, maintaining social responsibilities [23]. Mobility restriction, which may inhibit physical activity, human-computer interaction and technology usability, is a core functional impairment associated with the health, quality of life and participation of older people [24, 25].

A second potentially enabling or disabling factor for out-of-home participation is perceived risk. Falling is a commonly perceived risk in old age, and fear of falling has been identified as a leading predictor of falling [26]. There is conflicting research about the locations of falls among older people, but research suggests that approximately half of all falls occur outside the home e.g. in the street or public space [27]. Despite this, most assessments and interventions to mitigate the risk of falls occur in the home environment, while little research has addressed how the risks that a person perceives might exist or arise during out-of-home participation [28]. Earlier studies show that other types of risks may be associated with out-of-home participation among older people, including perceived risks of getting lost, feeling stressed or embarrassed, which may be exacerbated by ET use [29].

Thus, the aim of this study was to investigate the ways in which perceived risks and ET use are associated with out-of-home participation, among older people in the UK. The following research questions were used to address the aim.

### Research questions

1. What patterns of out-of-home participation and Everyday Technology (ET) use can be found among the UK sample of older people?
2. How is Everyday Technology (ET) use associated with out-of-home participation among the sample?
3. How are perceived risk and other factors e.g. having a functional impairment or access to a concession travel pass (CTP), associated with out-of-home participation among the sample?

## Methods

### Design and setting

A cross-sectional study design was used. Interviews were undertaken with 128 participants across five research sites (two sites in the London region, two sites in the Cumbria region, one site in Greater Manchester), in urban and rural regions of the UK. The geographical areas were chosen to enable an investigation of out-of-home participation and Everyday Technology use across different urban and rural environments of the UK. This justification is based on research that shows technology use can vary for older people living in rural or urban environments [30].

### Participants

Participants consisted of 128 older people aged 55 years or over. For the purposes of this study, there was no obvious reason to use the traditional age cut-off of 65 years. Rather, there is a need to develop more insights into the consequences of dementia and functional impairments for those that are not yet retired and who are identified by themselves and their environment as old people. Research has shown that participation in social activities is a modifiable risk factor for developing dementia [31]. By including people from 55 years old, the study may also contribute to the field of health promotion and dementia prevention. The sample therefore included older people living with a diagnosis of dementia (*n* = 64) as well as older people with no known cognitive impairment (*n* = 64) (Table 1). Participants with dementia had a diagnosis of dementia in the mild stage, given by a physician [33, 34]. Participants with dementia were recruited through the National Healthcare Service (NHS) e.g. memory clinics, in addition to local community-based groups e.g. memory cafes, and local Alzheimer Associations. Older adults without known cognitive impairment were recruited through local networks e.g. community-based activity or social groups. Participants were recruited according to the following inclusion criteria: (i) aged 55 years or over; (ii) ability to consent to the decision to take part in the research themselves; (iii) living in ordinary housing in the community; (iv) to some extent, undertaking activities and participating in at least one place outside home independently or with support; (v) being a user of at least some ET; (vi) managing without any vision or hearing limitations which could not be compensated via technical aids; and (vii) living without any other condition such as Multiple Sclerosis, that may impact the person’s participation and use of ET.

**Table 1:** Characteristics of participants

Measure	Participants (*n* = 128)
Gender, n (%)	
Female	63 (49.22)
Male	65 (50.78)
Age	
	Median: 76.00 IQR: 68.25–82.00
	Min-Max: 55.00–96.00
Dementia diagnosis, n (%)	
Dementia	64 (50.00)
No known cognitive impairment	64 (50.00)
MoCA^a^	
	Median: 24.00 IQR: 21.00–26.00
	Min-Max: 12.00–30.00
Years of education	
	Median: 12.00 IQR: 11.00–14.00
	Min-Max: 7.00–21.00
Living arrangement, n (%)	
Cohabit	79 (61.72)
Live alone	49 (38.28)
Living environment, n (%)	
Urban	98 (76.56)
Rural	30 (23.44)
Drive a car, n (%)	
Driver	72 (56.25)
Non-driver	56 (43.75)
Concession travel pass, n (%)	
Concession travel pass	68 (53.12)
No concession travel pass	60 (46.88)
Functional impairment, n (%)	
Functional impairment	110 (85.94)
No functional impairment	18 (14.06)
Everyday Technology use (*n* = 49)	
	Median: 16.00 IQR: 9.00–22.00 Min-Max: 1.00–35.00
Perceived risk of falling, n (%)	
Perceived risk	56 (43.75)
No perceived risk	72 (56.25)
Perceived risk of getting lost, n (%)	
Perceived risk	23 (17.97)
No perceived risk	105 (82.03)
Perceived risk of feeling embarrassed^b^, n (%)	
Perceived risk	35 (27.56)
No perceived risk	92 (72.44)
Perceived risk of feeling stressed, n (%)	
Perceived risk	41 (32.03)
No perceived risk	87 (67.97)

^a^Montreal Cognitive Assessment has possible scores from 0 to 30. A higher score indicates higher cognitive status [32]

^b^Missing data (data for one participant missing)

### Data collection procedures

The interviews were performed by two registered occupational therapists (SNG and SW). The interview was comprised of four stages: (i) the Participation in ACTivities and Places OUTside the Home Questionnaire (ACT-OUT) [5]; (ii) the Montreal Cognitive Assessment (MoCA) [32]; (iii) a demographic questionnaire; (iv) the Everyday Technology Use Questionnaire (ETUQ) [10]. No formal power calculation was used due to the exploratory design of the study however power calculations can be generated based on the findings of this study for subsequent research. Written and verbal informed consent was obtained from each participant prior to data collection. To mitigate against fatigue, interviews occurred at the participant’s home or another preferred location and were adapted to the needs of each participant e.g. spread over one to three sessions, with each session lasting no longer than 90 min.

### Measure: dependent variable

#### Out-of-home participation

The objective of the Participation in ACTivities and places OUTside home Questionnaire (ACT-OUT) is to capture detailed information on places and activities *in combination*, specifically identifying participation restrictions and pointing out barriers and facilitators in different contexts [5]. For the purposes of this study, the ACT-OUT was used to investigate out-of-home participation in places. Detailed information about the development of the ACT-OUT and the functioning of its rating scale can be found in earlier research [5]. Psychometric testing of the ACT-OUT is ongoing. In the ACT-OUT, places are defined according to four domains: (A) places for purchasing, administration and self-care [*n* = 6]; (B) places for medical care [*n* = 5]; (C) places for social, spiritual and cultural activities [n = 6]; (D) places for recreation and physical activity [*n* = 7]. The dependent variable (out-of-home participation) measures present participation as reported by the participants (out of a count of 24 places). In order to facilitate a conservative interpretation of the analysis, this count was analyzed according to quartiles: Quartile 1 (1–12 places); Quartile 2 (13–16 places); Quartile 3 (17–18 places); and Quartile 4 (19–24 places). Division of the dependent variable (outcome) according to quartile cut-points is used in research to promote interpretation of the clinical significance of the dependent variable [35]. This is particularly useful in exploratory research, for instance using new assessment tools, where theoretical or clinical justification for cut-points is not yet available but where a more nuanced interpretation without the loss of power associated with median dichotomization is required [35, 36].

### Measures: independent variables

#### Everyday technology use

The Everyday Technology Use Questionnaire (ETUQ) identifies the perceived level of difficulty experienced when using 90+ ET items [10]. Information about the ETUQ’s rating scale and its psychometric properties are described in earlier studies [16, 37, 38]. The ETUQ captured use of ET, comprised of a number of ET which can be used to engage in activities outside home (*n* = 16) e.g. ATMs, train ticket machine, as well as ‘portable ET’ that can be used both at home and outside home (*n* = 33) e.g. Smartphone, Hearing aid, Tablet. ‘Domestic ET’ which are typically used for activities performed in the home environment (*n* = 39) e.g. Kettle, Oven, or Lawnmower were excluded due to the focus on out-of-home participation [39]. The outcome generated from the ETUQ was first dichotomized according to if the ET was used (1) or not used (0) and then summed up per participant giving a possible total of 0 to 49.

#### Access to a concession travel pass (CTP)

The demographic questionnaire was used to gather information for the analysis of results with respect to a range of relevant contextual and person-related factors (Table 1). Participants reported a dichotomous answer of yes or no to currently having access to a CTP.

#### Functional impairment

According to the demographic questionnaire, functional impairment was self-reported by participants. If more information about a person’s functional impairment came to the fore in other parts of the interviews this was noted. Functional impairments included vision or hearing impairment (which could be compensated via technical aids e.g. glasses), reduced fine motor function, reduced walking ability, reduced arm function, and medical diagnoses (e.g. diabetes). Functional impairment was in addition to dementia for those living with a dementia diagnosis. Responses were dichotomized based on the participant reports of having one or more functional impairments, or no functional impairment.

#### Perceived risk outside the home

Participants responded to four Likert-scale questions in the ACT-OUT regarding how concerned they were about different types of risk whilst participating in places outside home (very concerned; concerned; unconcerned; very unconcerned). The four types of perceived risk were (i) falling; (ii) getting lost; (iii) feeling stressed; (iv) feeling embarrassed. Responses were dichotomized according to either perceived risk (very concerned; concerned) or no perceived risk (unconcerned; very unconcerned).

### Data analysis

The data were shown not to be normally distributed according to normality tests (Kolmogorov-Smirnov and Shapiro-Wilk tests) undertaken in the Statistical Package for Social Sciences (SPSS) computer software, version 26 [40]. To explore the patterns of out-of-home participation (Research Question 1), descriptive statistics including hierarchies of counts were used e.g. patterns of abandoned or retained participation. A count of total participation according to each type of place in the past was subtracted from a count of total participation in each type of place in the present. The difference between the total counts of participation in the past and the present indicated a change in total participation for each type of place. Due to the non-normally distributed continuous data, non-parametric tests were used. The strength of associations was determined using Cohen’s guidelines for social sciences: .1–.3 (small); .3–.5 (medium); and .5–1.0 (large) effect [41]. The alpha level was set to .05 for all analyses.

Research Questions 2 and 3 were investigated through ordinal regression. Ordinal regression was used to identify the association between the ordinal levels of the dependent variable (out-of-home participation) and the independent variables (ET use, access to a CTP, having a functional impairment, and perceived risk of falling outside home). Associations are reported according to log-adjusted regression coefficients (odds ratio), the estimate of the effect with confidence intervals, and significance is indicated (Table 2). To support the clinical relevance of the findings, interpretation of the probability of a person having a higher level of out-of-home participation is based on five technology items for the ET use variable (as opposed to one technology item). Ordinal regression is applied in a similar way to standard logistic regression with the exception of using ordinal levels of participation instead of a dichotomous dependent variable [42]. No collinearity was found among the independent variables except for collinearity found between having a diagnosis of dementia and ET use. Due to the focus of the research questions on ET use, diagnosis of dementia was not included as an independent variable. Testing for proportional odds was used to evaluate the homogeneity of the effects across categories of the dependent variable.

**Table 2:**
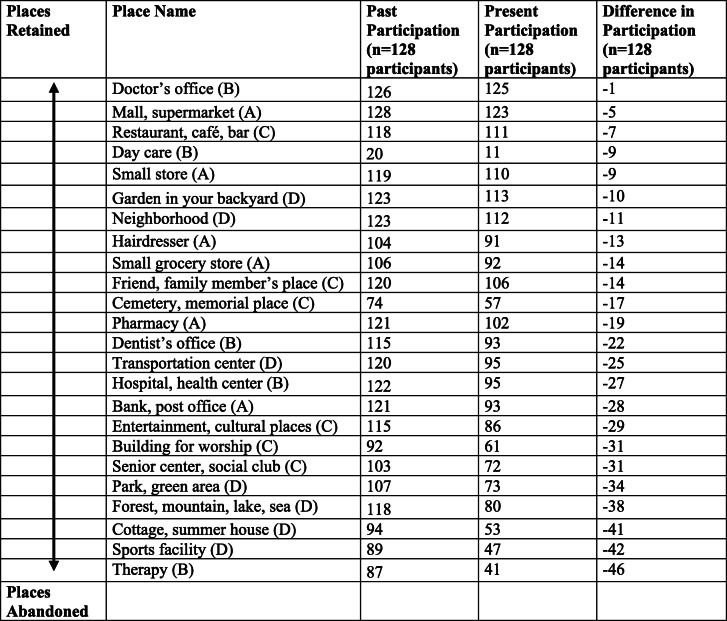
Ordinal regression model (dependent variable: out-of-home participation)

Key: A: Consumer, administration and self-care places; B: Places for medical care; C: Social, spiritual and cultural places; D: Places for recreation and physical activities

## Results

### Description of participants

Table 1 summarizes the demographics of the participants, in addition to the independent variables. The median age of participants was 76.00 (IQR: 68.25–82.00, range: 55.00–96.00). A higher percentage of participants were drivers (56.25%) compared to non-drivers (43.75). Similarly, a higher percentage of participants reported having a CTP (53.12%) than participants without access to a CTP (46.88%). The majority of participants (85.94%) reported having some type of functional impairment. The most common type of concern was the perceived risk of falling outside home, which was reported by 43.75% of the sample. The least common type of concern was the perceived risk of getting lost which was reported by 17.97% of the sample. The perceived risk of feeling stressed outside home was reported by 32.03% of the sample and the perceived risk of feeling embarrassed outside home was reported by 27.56% of the sample.

### Patterns of out-of-home participation and everyday technology use

Table 3 shows counts of out-of-home participation in places in the past and present, among the sample. A pattern of abandonment was found among the types of places used for recreation and physical activities (Domain D, e.g. sports facility; cottage, summer house; forest, mountain, lake, sea; park, green area) as well as social, spiritual and cultural places (Domain C, e.g. senior center, social club; building for workshop; entertainment, cultural places). The types of places that older people retained over time were more varied, tending to be those places for medical care (Domain B e.g. Doctor’s surgery) or consumer, administration and self-care places (Domain A e.g. Mall, supermarket). There was however no clear retention pattern, older people reported continuing to participate in other places such as those for social, spiritual and cultural places (Domain C e.g. restaurant, cafe, bar).

**Table 3:** Ordinal regression model (dependent variable: out-of-home participation)

Independent Variable	B	SE	Exp (B) Odds ratio	95% CI for Exp (B)	Wald	*p*
ET Use^a^	.080	.020	1.083	(1.041, 1.127)	15.455	***
Perceived Risk of Falling	.916	.359	2.499	(1.235, 5.053)	6.491	*
Concession travel pass	1.372	.354	3.943	(1.970, 7.893)	15.006	***
Functional Impairment	−.754	.487	.470	(.181, 1.223)	2.395	

*p < .05, **p < .01, ***p < .001

Nagelkerke *R*^*2*^: 0.32

^a^ET Use odds ratio refers to 5 technological items = 1.492

**Fig. 1:**
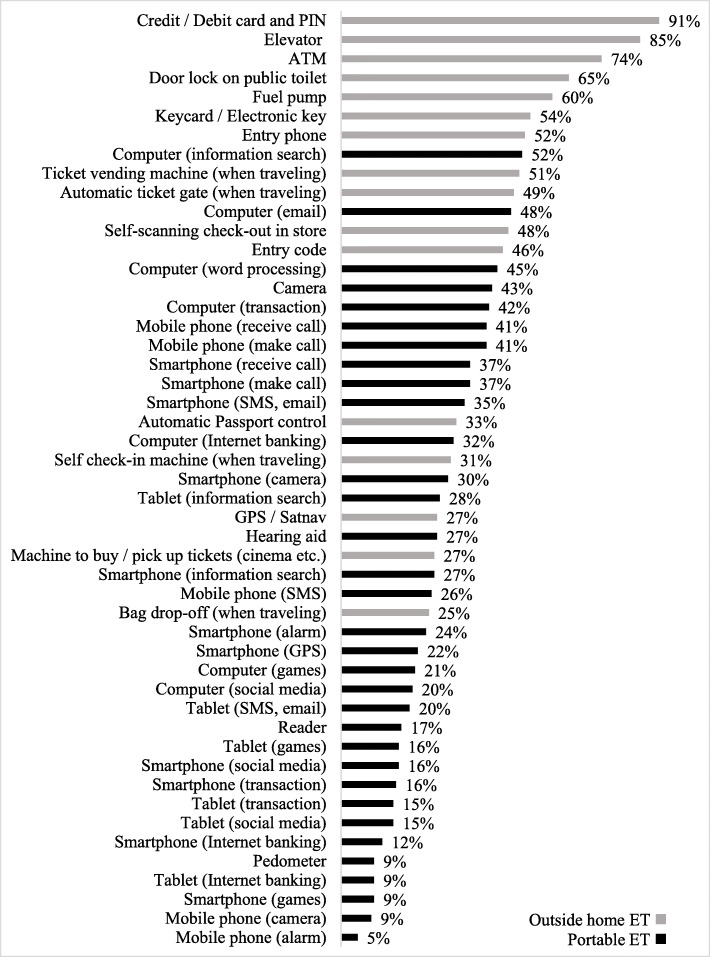
Hierarchy of highest to lowest percentages of participants’ Everyday Technology (ET) use

The median total amount of ET use was 16.00 (Inter-quartile range [IQR]: 9.00–22.00, min-max: 1.00–35.00) out of a total of 49.00 (Table 1). According to the percentages of counts of ET use (Fig. 1), the type of ET used to a lesser degree included ‘portable ET’ which can be used at home and outside home e.g. Mobile phone using the alarm and camera functions; Smartphone using the games function; Tablet for internet banking; and Pedometer. Conversely, the type of ET used to a higher degree tended to be ET used only outside home e.g. Card/Debit card and PIN; Lift; ATM; Door lock on public toilet; and Fuel pump.

### Associations between everyday technology use and out-of-home participation

Univariate analysis showed that ET use was associated with a 1.507 higher probability (95% CI: 1.045–1.129, *p* < .001) of a person having a higher level of out-of-home participation.

### Perceived risk and out-of-home participation

Univariate analysis indicated non-significant associations between three of the four risk variables and the ordinal levels of the dependent variable (out-of-home participation): (i) getting lost (OR: .624, 95% CI: .276–1.412, *p* = .257); (ii) feeling stressed (OR: 1.196, 95% CI: .613–2.333, *p* = .601); (iii) feeling embarrassed (OR: .856, 95% CI: .424–1.723, *p* = .662). However, a significant association was identified for perceived risk of falling, therefore a higher probability of perceived risk of falling outside home (OR: 3.582, 95% CI: 1.842–6.966, *p* < .001) was associated with a higher level of the dependent variable (out-of-home participation).

### Perceived risk, other factors and out-of-home participation

Based on the ordinal levels of the dependent variable (out-of-home participation), twenty-two participants (17.19%) reported participation in Quartile 1 (1–12 places). Forty-two participants (32.81%) reported participation in Quartile 2 (13–16 places) and thirty-five participants (27.34%) reported participation in Quartile 3 (17–18 places). Twenty-nine participants (22.66%) reported participation in Quartile 4 (19–24 places). ET use was associated with a 1.492 higher probability (95% CI: 1.041–1.127, *p* < .001) of a person having a higher level of out-of-home participation, given that the other variables were controlled for. A perceived risk of falling outside home was associated with a higher probability of a person having a higher level of out-of-home participation (OR: 2.499, 95% CI: 1.235–5.053, *p* = .011). Having access to a CTP was associated with a higher probability of a person having a higher level of out-of-home participation (OR: 3.943, 95% CI: 1.970–7.893, *p* < .001). However, having a functional impairment was associated with a low probability of a higher level of out-of-home participation (OR: .470, CI: .181–1.223, *p* = .122). This association was not statistically significant although it indicated that having a functional impairment, which may be in addition to dementia for those with a dementia diagnosis, may be associated with a lower probability of a person having a higher level of out-of-home participation. The proportional odds testing was non-significant indicating that the assumption of proportional odds was met.

## Discussion

The main contribution of this study is that it demonstrated ways in which perceived risks and ET use were associated with out-of-home participation, a sample of older people in the UK. In order to address the aim, the study’s first research question investigated patterns of out-of-home participation and ET use among the sample. Across the sample of older people, a pattern of abandonment was found among the types of places participated in the past compared to the present, these tended to be social, spiritual and cultural places (Domain C e.g. senior center, social club; building for workshop; entertainment, cultural places) as well as places used for recreation and physical activities (Domain D e.g. sports facility; cottage, summer house; forest, mountain, lake, sea; park, green area) (Table 3). The pattern of abandonment was shared by older people with and without dementia, which corroborates earlier research that showed commonalities in patterns of participation by both older people with and without dementia in Sweden and Switzerland ([39], Margot-Cattin et al., unpublished observations). This has clinical significance because the study’s identification of the abandonment of specific place types may help to develop more targeted health and social care interventions, in addition to providing evidence for the types of places which require adaptations to enable participation for older people.

Patterns were also identified according to ET use. The findings provide insight into the types of ET that the sample of older people used in relation to out-of-home participation. Not surprisingly, Fig. 1 demonstrated that ET typically used outside home (e.g. Credit/ Debit card and PIN; Lift; ATM; Door lock on public toilet; Fuel pump) were used to a higher degree than ‘portable ET’ which can be used at home or outside home (e.g. Mobile phone (alarm); Mobile phone (camera); Smartphone (games); Tablet (internet banking); Pedometer). Due to the exploratory nature of the study, investigation of the motivation for differences in ET use was not explored although a review of the literature about ET use provides several reasons for why older people may use ET outside home to a higher degree than ‘portable ET’. Reasons include, a preference for ET perceived as useful or essential to the performance of purposeful activities of daily living (e.g. using a debit card to make a financial transaction) [15, 43]; social inequity, inaccessibility and expense of ET (e.g. ET used outside home are typically free to use, do not require personal ownership) [44]; and increased familiarity with specific types of ET (e.g. users of ET outside home can receive external information, observe others using the ET, and imitate their actions) [45]. By comparison older people have reported a degree of distrust and a lack of familiarity for newer, ‘portable ET’ (e.g. Tablets and Smartphones) which require regular updates as well as featuring personal data tracking or monitoring devices [15, 46]. Existing literature underlines the emergence of a ‘digital underclass’ of older people [47] although this does not provide information about the broader use of ET among older people participating in places outside home. Subsequent research is needed to explore motivators and inhibitors in relation to ET use for out-of-home participation, among a “digital underclass” of older people.

Regarding research question two, an association was found between out-of-home participation and ET use which suggests that higher use of ET is associated with higher probability of higher out-of-home participation. The findings reinforce earlier research that showed ET use is related to activity involvement among people with mild cognitive impairment in Sweden [14]. More specifically, this study contributes to the knowledge base by demonstrating that the association between ET use and out-of-home participation is evident among different aging populations (older people living with and without dementia) and across different contexts (urban and rural regions of the UK). It is not yet known whether higher levels of out-of-home participation ensure that a person is exposed to more ET and therefore a person uses more ET and develops a higher ability to use ET. Or conversely, whether increased ET use and an accompanied higher ability to use ET, may enable greater opportunities to participate. Earlier research focused on dimensions of internet use revealed a positive association between higher variety of internet use and increased amount of internet use [48]. It is important to build on such knowledge for ET in general because living within an increasingly technological society ensures that ET use, including ICT, impacts social and spatial dimensions [49] as well as practices and routines in the everyday lives of older people [7]. For those who are able, and choose to use ET, it may be a mitigating factor against social isolation. However, for other older people who may not be able, or choose not to use ET including ICT, it can present as a contraindication to their participation in society and exacerbate the risk of social isolation and loneliness [50]. Further research is required to understand the association between ET use and ability to use ET, in relation to out-of-home participation in places as well as activities.

Finally, in accordance with the third research question, the findings show that the variable of ET use may only partially explain out-of-home participation. This compelled the investigation of other enabling or disabling factors in relation to out-of-home participation. Perceived risk of falling was associated with the probability of a higher level of out-of-home participation. The findings suggest that the more a person participates in places outside home, the more they may encounter the risk of falling. This differs from earlier research which associates fear of falling with avoidance behaviors, including reduced engagement in daily activities. For instance, Jefferis et al., [51] found that within a cohort of 1680 men, those who experienced recurrent falls or were fearful of falling engaged in lower daily activity levels. The concept of perceived risk of falling used in this study may differ from the more commonly used concept of fear of falling. Further qualitative research would be insightful in order to explore what perceived risk of falling means for older people with and without dementia whilst participating in places and using ET outside home. All other types of perceived risk (getting lost; feeling embarrassed; feeling stressed) were not significantly associated with out-of-home participation.

The findings revealed a non-significant association between out-of-home participation and having a functional impairment. Earlier research has demonstrated that living with some form of functional impairment can negatively influence a person’s ability to perform a range of different activities of daily living to some degree [23]. However, an inference from the findings is that older people living with a functional impairment may be facilitated to participate outside home by having a CTP. Access to a CTP (e.g. the Transport for London Freedom Pass) was associated with a higher probability of a higher level of out-of-home participation. Access to a CTP whether due to age or disability enables the use of, and access to, effective and affordable public transportation. Increased use and access to public transportation can enable all people, including older people, to participate in activities of daily living for freedom of movement as well as health benefits such as increased social engagement [20], physical activity [19] and the maintenance of one’s quality of life and autonomy [52, 53], which is also empirically supported in the findings from this study.

ET is central to the access and use of public transportation. The ability to use ET e.g. ticket machines, electronic travel passes, automated ticket gates, journey planning apps and GPS etc. can be a facilitator or barrier for accessing public transport in order to participate in society. Whilst ET such as ticket machines or GPS applications require relatively complex cognitive, perceptual and fine motor processes, the automated system of a CTP does not generally require the user to reload or activate their pass manually. This may help the older traveler to travel ‘freely’ without requiring the cognitive processes to manage the travel pass. The need to promote accessible public transportation is highlighted in policies such as the UN (2015) Sustainable Development Goals [9] and the WHO’s (2007) agenda on Age-Friendly Cities [54], based on the hypothesis that without transportation, or an effective means of supporting people to meet and connect, other facilities and services intended to promote health and wellbeing are rendered inaccessible. The findings underline the complexity of promoting out-of-home participation using public transportation because whilst access to a CTP was a contributing factor to participation, a variety of other factors were associated with participation, including the use of ET, perceived risk outside home and having a functional impairment.

### Methodological considerations

Due to the exploratory nature of the study utilizing a relatively new assessment tool (ACT-OUT), the study is not without limitations. Whilst the ETUQ is a questionnaire validated for use with older people living with and without dementia [16, 37, 38], the ACT-OUT Questionnaire is a new and therefore unvalidated questionnaire. Psychometric testing of the ACT-OUT Questionnaire is underway, including a forthcoming content validity index. It is however important to undertake research using new assessment tools which report the needs of the older person themselves, particularly among persons with dementia who are underrepresented in research [55]. This also justifies the decision to emphasize self-report over proxy-reporting or observational assessment of the older person’s functional impairment, due to the focus on the person’s perceived functional impairments whilst engaging in out-of-home participation.

The study was potentially limited by the small sample size. One measure to ameliorate reduced power size was to use quartile cut-points, in favour of a dichotomous cut-point which can inflate the margin of error [35, 36]. Findings from this exploratory study may be valuable for subsequent confirmatory analyses using a larger sample size. However, due to the current sample size of this study generalizability of the findings cannot be assumed. In addition, the representativeness of the sample was potentially compromised by the recruitment strategy, in two ways. First, recruitment of participants was undertaken using convenience sampling. Use of a sampling frame instead of convenience sampling may have yielded a more systematic approach to sampling participants from both urban and rural geographical locations in the UK. Second, recruitment of participants with and without dementia arose from different sources which may account for differences within the sample. All participants were older people living in their communities however for this study there was an ethical requirement to recruit older people with a dementia diagnosis across the five research sites in the UK via the NHS.

The study sought to investigate out-of-home participation among older people living in their own homes in the community and research indicates that this includes people living with dementia, in the mild stage. Whilst older people are associated with ‘noise’ in terms of comorbidity or polypharmacy, to restrict studies to ‘healthy’ older people would limit the external validity of the findings [55]. Therefore, the study sought to include older people with and without dementia although diagnosis was not the focus of the study. An additional rationale for analyzing the sample as a whole, as opposed to splitting the sample according to diagnostic groups, is due to collinearity found between the ET use and diagnosis variables. ET use was a focus of the study aim and research questions and thus it was emphasized. Prior research has shown that ET use is a complex phenomenon influenced by numerous factors and not only diagnostic severity [13].

### Implications for policy and practice

The study provides a novel contribution to the discourse on out-of-home participation and ET use for older people, based on at least three points. Firstly, whilst the diagnosis of dementia is acknowledged, the study also explores how functional health relates to out-of-home participation. Secondly, the potential enabling influence that having access to a CTP can have on the out-of-home participation of older people is emphasized. Thirdly, a few studies have explored ET use and participation in activities of daily living however this study differs in its focus on the risks that older people perceive whilst participating outside home.

## Conclusion

The study identified patterns of out-of-home participation and ET use among a sample of older people with and without dementia in the UK. Potential clinical insights may be gleaned from identification of patterns, such as the tendency for older participants to use outside home ET (e.g. credit/debit card payments, elevator, ATM) to a higher degree than portable ET (Smartphones or mobile phones). The statistically significant association found between ET use and out-of-home participation suggests that ET use may be a clinically significant consideration for more targeted health and social care planning, among older people living with and without dementia in their communities, however this requires further research. Furthermore, perceived risk of falling, having a functional impairment and, access to a CTP were found to be factors for out-of-home participation and this indicates a potential opportunity to enable out-of-home participation by acknowledging these factors among older people with and without dementia in the UK.

## Data Availability

The ethical permissions have enabled the analysis of the research data for the purposes of this study but not for the publication of all data for other purposes.

## Abbreviations

ACT-OUT: Participation in ACTivities and Places OUTside the Home Questionnaire
CI: Confidence interval
ET: Everyday Technology
CTP: Concession travel pass
ETUQ: Everyday Technology Use Questionnaire
ICT: Information and Communication Technology
IQR: Inter-quartile range
MoCA: Montreal Cognitive Assessment
NHS: National Health Service
OR: Odds ratio
